# Poster 2002: Transfer factor may provide immunomodulation in cutaneous tuberculosis

**DOI:** 10.1186/1939-4551-7-S1-P19

**Published:** 2014-02-03

**Authors:** Laura Vidal, Damian Palafox

**Affiliations:** 1 Allergy and Immunology Department. Respiratory and Allergic Diseases Unit Xalapa, Mexico; 2 Hospital General Gea Gonzalez. Mexico, Mexico

## Background

Transfer factor is a Dialyzable Leukocyte Extract. It has been widely used in several clinical scenarios such as primary immunodeficiencies, infectious diseases and malignancies with satisfactory results. One of the mechanisms of action proposed is the enhancement of the cellular immunity, however this have not yet been clarified.

## Methods

We tested transfer factor in a 1 year old and 3 months patient diagnosed with Ganglionar Tuberculosis. 1 week after the administrarion of the Bacillus Calmette-Guérin vaccination, the present developed fever, cervical, submandibular, supraclavicular, inguinal and axillary lymphadenopathy. Later on the patient devoloped cutaneous clinical manifestations of tuberculosis such as scrofuloderma, fistulas, hypertrophic scars and ultimately, queloids. The patient had previously undergone short-term strictly supervised treatment for tuberculosis with very poor results. When the treatment was first administered, the patient had the following data: Total White Blood Count 12.9 Lymphocytes: 29% (12–46) CD3: 26.3% (59–90) T helper Cells (CD3/CD4) 21.6% (42–58) Cytotoxic T cells (CD3/CD8) 5.1% (17–33) Natural Killer Cells (CD56) 2.1% (3–7) B cells (CD19) 67.6 % (0–10).

## Results

At the end of the treatment with Transfer Factor the patient's immune system was enhanced in terms of cell count and improval of skin manifestations. Total White Blood Count 6.5 Lymphocytes: 51.3% CD3: 48.5% T helper cells(CD3/CD4) 31.2% Cytotoxic T Cells (CD3/CD8) 14.6% Natural Killer cells (CD56) 12.2% B cells (CD19) 985%. Cicatrization process was improved, with involution of skin lesions os scrofuloderma and fistulas. Lymphadenopathy was no longer encountered. We have followed the patient for a year and half and no relapses have been encountered.

## Conclusions

Transfer Factor may have immunomodulatory properties and have the potential to be a valuable option as adyuvant therapy in cases of ganglionar and cutaneous tuberculosis refractory to conventional treatments. To our knowledge, as we have previously stated in a WAO forum, this is the first report of a case of the disease treated satisfactorily with transfer factor.

